# Supplementary material for: Determinants of non-Hodgkin’s lymphoma at Felegehiwot specialized hospital, North West Ethiopia: A case-control study
Source: PLoS One. 2020 Dec 28;15(12):e0243561. doi: 10.1371/journal.pone.0243561 (PMC7769477; doi:10.1371/journal.pone.0243561)
Supplement: S1 File — (PDF) [file pone.0243561.s001.pdf]

English Version Questionnaire

Part I: Socio-demographic conditions; circle the answer after reading carefully

Questions	Response
Age of the study participant	__________years
Sex of the study participant	1. Male<br>2. Female
Residence of the study participant	1. Urban<br>2. Rural
Religion of the study participant	1. Orthodox<br>2. Muslim<br>3. Protestant<br>4. Others --------------
Ethnicity	1. Amhara<br>2. Non-Amhara
Educational status	1. Unable to read and write
	2. Elementary
	3. Secondary
	4. Diploma and above
Occupation	1. Farmer
	2. Housewife
	3. Government employee
	4. Military

	5. Factory worker
	6. Day labour
	7. Others, if any specify ---------------------
Marital status	1. Single
	2. Married
	3. Divorced
	4. Widowed
Monthly income (in Birr)	-----------------_________________

Part II: - NHL cancer exposure condition; circle the answer after reading carefully

Questions	Responses
Did you have experience about Physical exercise/sport in your life?	A. Yes<br>B. No
what is the condition (stage) of the disease during contact patient?	A. Stage one<br>B. Stage two<br>C. Stage three<br>D. Stage four
what was your occupation in the past__________________?	-------------------------
Were there any chemical exposure in the past?	A. Yes<br>B. No

if you were exposed to chemicals, by what kind of chemicals you exposed?	A. Pesticides<br>B. Phenoxyacetic acid<br>C. Herbicides<br>D. Organic Solvents<br>E. Benzene<br>F. DDT
For how long do you exposed for these types of chemical?	A. for less than five years<br>B. for more than seven years<br>C. for more than ten years<br>D. for more than fifteen years
Do you have experience about Smoking Cigarette?	A. YES<br>B. NO
What was your experience?	A. Ex-smoker<br>B. Current smoker<br>C. Non -smoker
If you are current smokers, for how many years you smoke?	A. 2-4 years<br>B. 5- 7years<br>C. 8-10years<br>D. >10 years
Do you drink alcohol?	1. Yes<br>2. No

If your answer is ‘Yes’, what types of Alcohol were taking?	A. Beer<br>B. Cultural alcohol<br>C. Wine<br>D. Others
Were there any diseases other than cancer before?	A. Yes<br>B. No
if your answer is ‘yes’, what was the diseases	A. HIV/AIDS<br>B. Diabetes Mellitus<br>C. Hypertension<br>D. Cardiovascular<br>E. Others, if any specify-------------------------
Do you drink coffee?	A. Yes<br>B. No
If your answer is ‘Yes’, how many years permanently drink coffee?	A. >10 years<br>B. 10-20 years<br>C. 21-30 years<br>D. >30 years
Nutritional/ feeding/ practice in the past	A. Meat<br>B. Vegetable<br>C. Non-vegetable<br>D. Others, if any specify---------------

was there a history of NHL Cancer of before?	A. Yes<br>B. No<br>C. Unknown

Part III: NHL cancer (diseases) condition and treatment related questions; circle the answer after reading carefully

Questions	Responses
When was the disease started?	A. 1-6 months<br>B. 6 months up to 1 year<br>C. 1 up to 2 years<br>D. > 2 years<br>E. Not mentioned
Have you got any treatment?	A. Yes<br>B. No
If your answer is 'Yes', what kind of treatment you have got?	A. Chemotherapy only<br>B. Radiotherapy only<br>C. Both chemotherapy and Radiotherapy
What is the outcome, if the patient was treated?	A. Progressed/improved<br>B. Deteriorated<br>C. Same
The weight of the study participant? (in kilogram)	-------------------

What was the height of the study participant? (in meter)	-------------------
The BMI of the study participant	-------------------
was there history cancer other than NHL cancer before?	A. Yes<br>B. No<br>C. unknown
Where are you treated during started the disease?	A. Cultural medicine<br>B. Modern medicine<br>C. Holly water<br>D. Unknown
What kind/route of medication you took?	A. PO (taken in mouth)<br>B. Intravenous<br>C. Intra medullar<br>D. Unknown
If you took the medication, the duration of Treatment was?	A. three weeks<br>B. four weeks<br>C. five weeks<br>D. $\geq$ sex weeks
