# Supplementary material for: Determinants of non-Hodgkin’s lymphoma at Felegehiwot specialized hospital, North West Ethiopia: A case-control study
Source: PLoS One. 2020 Dec 28;15(12):e0243561. doi: 10.1371/journal.pone.0243561 (PMC7769477; doi:10.1371/journal.pone.0243561)
Supplement: Attachment — Submitted filename: Response to Reviewers.docx [file pone.0243561.s003.docx]

Getasew Mulat Bantie, BSc, MPH

Lecturer, Department of Public Health,

Faculty of Community Health,

Alkan Health Sciences, Business and Technology College,

Bahir Dar, Ethiopia

Email: [getasewmulat@gmail.com](mailto:getasewmulat@gmail.com)

Dr. Yan Li

Academic Editor, PLoS One

October 05, 2020

Dear Dr. Yan Li,

My colleagues and I are happy to re-submit to you a revised version of our manuscript PONE-D-20-14290, ‘Determinants of Non-Hodgkin’s Lymphoma at Felegehiwot Specialized Hospital, North West Ethiopia: a case-control study*’* for *PLoS One*. Thank you for giving us the opportunity to revise and re-submit this manuscript. In keeping with your last message, I am re-submitting this revision on the stated deadline, November 03 2020.

We thank the reviewers for their detailed and thoughtful suggestions. We have incorporated the recommended changes in the manuscript to the best of our ability and we feel that our revised manuscript is significantly improved as a result of their advice.

We have responded specifically to each suggestion in the memo below and have highlighted our changes in the manuscript text. In the memo, portions of the reviewer’s comments are reproduced in italic text, and our responses to the reviewer’s' comments are listed beneath in normal text.

Thank you once again for your time and attention to our manuscript. We look forward to working with you and the reviewers to move this manuscript closer to publication.

Best regards,

Getasew Mulat Bantie

Editors and Reviewer Comments and Responses

*Academic Editor: Please ensure that your manuscript meets PLOS ONE's style requirements, including those for file naming*

We have revised the manuscript and our file names to conform with PLoS’s style requirements.

*Academic Editor: We suggest you thoroughly copyedit your manuscript for language usage, spelling, and grammar. If you do not know*

*anyone who can help you do this; you may wish to consider employing a professional scientific editing service.*

We acknowledge these inadvertent errors and have revised the manuscript to correct grammatical and typographical mistakes. These changes are highlighted throughout the document with track changes.

*Academic Editor: Please include additional information regarding the survey or questionnaire used in the study and ensure that you have provided sufficient details that others could replicate the analyses.*

Thank you very much for your information. We authors added the questionnaire and SPSS data as supporting information in the manuscript.

*Academic Editor: You indicated that you had ethical approval for your study. In your Methods section, please ensure you have also confirmed that your IRB waived the need for parental consent for minors aged 13-17 and how parental permission was determined. In the manuscript you state: "13-17 years old patients,’ assents secured solely from them with parental/guardian permission".*

Thank you very much for your critical review. We authors added the ethical approval statement in the clear and track changed manuscript.

*Academic Editor: We note that you have indicated that data from this study are available upon request. PLOS only allows data to be available upon request if there are legal or ethical restrictions on sharing data publicly.*

Thank you very much for your information. We authors added the questionnaire and SPSS data as supporting information in the manuscript.

*Academic Editor: In your revised cover letter, please address the following prompts:*

*a) If there are ethical or legal restrictions on sharing a de-identified data set, please explain them in detail (e.g., data contain potentially identifying or sensitive patient information) and who has imposed them (e.g., an ethics committee). Please also provide contact information for a data access committee, ethics committee, or other institutional body to which data requests may be sent.*

Thank you very much for your information. We authors added the questionnaire and SPSS data as supporting information in the manuscript.

*b) If there are no restrictions, please upload the minimal anonymized data set necessary to replicate your study findings as either Supporting Information files or to a stable, public repository and provide us with the relevant URLs, DOIs, or accession numbers. We will update your Data Availability statement on your behalf to reflect the information you provide.*

Thank you very much for your action.

*Academic Editor: Your ethics statement must appear in the Methods section of your manuscript. If your ethics statement is written in any section besides the Methods, please move it to the Methods section and delete it from any other section. Please also ensure that your ethics statement is included in your manuscript, as the ethics section of your online submission will not be published alongside your manuscript.*

Thank you very much for your critical review. We authors added the ethical approval statement in the clear and track changed manuscript.

## ‘**Ethics approval and consent to participate**

Ethical approval obtained from the Institutional Review Board of Bahir Dar University. Permission letter was obtained from the Amhara National, Regional state Health Bureau and Public Health Institute prior to the data collection period. We authors can assure that the Institutional Review Board of Bahir Dar University waived the need for parental consent for minority groups. Before collecting the data, for patients whose age 7-12 years written consent received from parents/guardians and assent of patients; and for 13-17 years old patients,’ assents secured solely from them with parental/guardian permission. For older than 17 years patients, consent received solely from them. The names of the patients did not use to ensure anonymity and confidentiality. All information obtained from the patients was kept confidential.’ (Page 8 of the manuscript)

**Review Comments to the Author**

*Reviewer #1: The authors desired to study epidemiologic risk factors for NHL in their hospital in West Ethiopia. The most provocative finding is the association of increased risk with chemical exposure. This remains an important global health questions.*

Thank you! *Reviewer #1: Abstract: Many places where parallel structure is needed to clean up the grammar. Many other grammatical errors throughout.*

Thank you very much for your critical comments! And we did amendments as per the given comments. (Page 2 -3 of the manuscript) *Reviewer #1: p.3 Background: Eliminate the first three paragraphs. Not all statements are accurate or relevant. Many are too rudimentary for a scientific paper in a major journal.*

Thank you very much for your critical review! We omitted the first three paragraphs as per the given comments. (Page 4 of the manuscript).
